# Oxidative damage and impairment of protein quality control systems in keratinocytes exposed to a volatile organic compounds cocktail

**DOI:** 10.1038/s41598-017-11088-1

**Published:** 2017-09-06

**Authors:** Marlène Dezest, Mickael Le Bechec, Laurent Chavatte, Valérie Desauziers, Benoît Chaput, Jean-Louis Grolleau, Pascal Descargues, Carine Nizard, Sylvianne Schnebert, Sylvie Lacombe, Anne-Laure Bulteau

**Affiliations:** 1 IPREM, UMR 5254, Université de Pau et des Pays de l’Adour, Hélioparc, 2 Avenue Pierre Angot, 64000 Pau, France; 2 Pôle RIME-C2MA, Ecole des mines d’Alès, Hélioparc, 2 Avenue Pierre Angot, 64053 Pau, France; 3 Service de chirurgie plastique et reconstructrice, CHU de Toulouse Rangueil, 1, avenue Jean-Poulhès, 31400 Toulouse, France; 4 Genoskin, Centre Pierre Potier, Oncopole, 31100 Toulouse, France; 5 LVMH Recherche. Life Science Department, 185 Avenue de Verdun, 45800 Saint Jean de Braye, France; 6 Institut de Genomique Fonctionnelle de Lyon (IGFL), Ecole Normale Superieure (ENS) de Lyon-CNRS- UMR 5242, 46 Allee d’Italie, 69364 Lyon, France

## Abstract

Compelling evidence suggests that volatile organic compounds (VOCs) have potentially harmful effects to the skin. However, knowledge about cellular signaling events and toxicity subsequent to VOC exposure to human skin cells is still poorly documented. The aim of this study was to focus on the interaction between 5 different VOCs (hexane, toluene, acetaldehyde, formaldehyde and acetone) at doses mimicking chronic low level environmental exposure and the effect on human keratinocytes to get better insight into VOC-cell interactions. We provide evidence that the proteasome, a major intracellular proteolytic system which is involved in a broad array of processes such as cell cycle, apoptosis, transcription, DNA repair, protein quality control and antigen presentation, is a VOC target. Proteasome inactivation after VOC exposure is accompanied by apoptosis, DNA damage and protein oxidation. Lon protease, which degrades oxidized, dysfunctional, and misfolded proteins in the mitochondria is also a VOC target. Using human skin explants we found that VOCs prevent cell proliferation and also inhibit proteasome activity *in vivo*. Taken together, our findings provide insight into potential mechanisms of VOC-induced proteasome inactivation and the cellular consequences of these events.

## Introduction

At the interface between the body and the environment, the skin is directly exposed to chemical oxidants, air pollutants all of which are potent inducers of reactive oxygen species (ROS)^1, 2^. Although, epidemiological and clinical studies highlight the adverse effects of pollution on human health, very little research is available to date concerning cutaneous effects^3^. A recent epidemiological study discovered a link between exposure to airborne particulate matter (PM), and the occurrence of skin aging, the detrimental effects were through the generation of ROS^4^. Volatile organic compounds (VOCs) are emitted from antropogenic and biogenic sources. Indoor air has been the focus of scientists over the last decade specifically because people spend most of their time indoors, at home, and sources of air pollution are numerous including building material, equipment, cleaning products and combustion processes like cooking^5, 6^. Many studies have focused on the airways and the lung that constitute preferential targets for gases^7^. VOCs have been shown to be irritant and to induce respiratory symptoms. Toluene, hexane and formaldehyde have been classified as priority indoor pollutants which are known to induce airway irritation and an inflammatory response^8–10^. Studies of indoor pollution effects have analyzed the effects of one single VOC. However, we have to take into account that people are exposed to a VOC cocktail and in our study, we consider the joint exposure to pollutants. Therefore, a synergic effect of each pollutant cannot be excluded. The exact mechanisms by which VOCs can cause skin damage has yet to be elucidated. Based on current evidence, there may be two potential mechanisms (i) generation of free radicals, (ii) induction of inflammatory response and disruption of the skin barrier^3^.

To study the effects of air pollutants with an *in vitro* model of human skin explants, new tools have to be developed using an air-liquid interface aimed at closely mimicking the physiological environment. The aim of the study was to design an adapted *in vitro* model using a direct device exposure for studying the cellular effects of 5 VOCs (hexane, toluene, acetaldehyde, formaldehyde and acetone) at doses mimicking low-dose chronic environmental exposure on skin keratinocytes and skin explants. Hexane and toluene toxicity is linked through their lipophilicity and accumulation in the lipid bilayer of cellular membranes leading to lipid peroxidation^11, 12^. Aldehydes produced during lipid peroxidation can lead to protein carbonylation and oxidation. The level of oxidatively modified proteins reflects the balance between free radical damage and proteolytic degradation. It is therefore important to investigate the response of proteases involved in the degradation of oxidized proteins due to VOC exposure. The current study was undertaken to characterize the effects of VOC exposure on proteasome activity and to assess how alterations in proteasome function may contribute to cell death. More than 80% of cellular proteins are degraded through this pathway including those involved in a broad array of processes such as cell cycle, apoptosis, transcription, DNA repair, protein quality control and antigen presentation^13^. Our results demonstrate that VOC exposure induced a significant decline in proteasome activity. This is accompanied by reduction in cell viability, apoptosis, accumulation of oxidatively modified protein, DNA damage and mitochondria dysfunction. Our results also demonstrated that exposure of keratinocytes to VOCs induced a significant decline in Lon protease activity, the protease in charge of protein degradation in the mitochondria. This was accompanied by mitochondrial ROS production. We also observed proteasome inhibition in human skin explants exposed to VOCs. Taken together, these findings suggest that protein quality control systems may be particularly vulnerable to inactivation in conditions associated with VOC exposure resulting in accumulation of oxidatively modified proteins, mitochondrial dysfunction and cell death.

## Results

### Effects of VOCs exposure on cell viability

**Figure 1:**
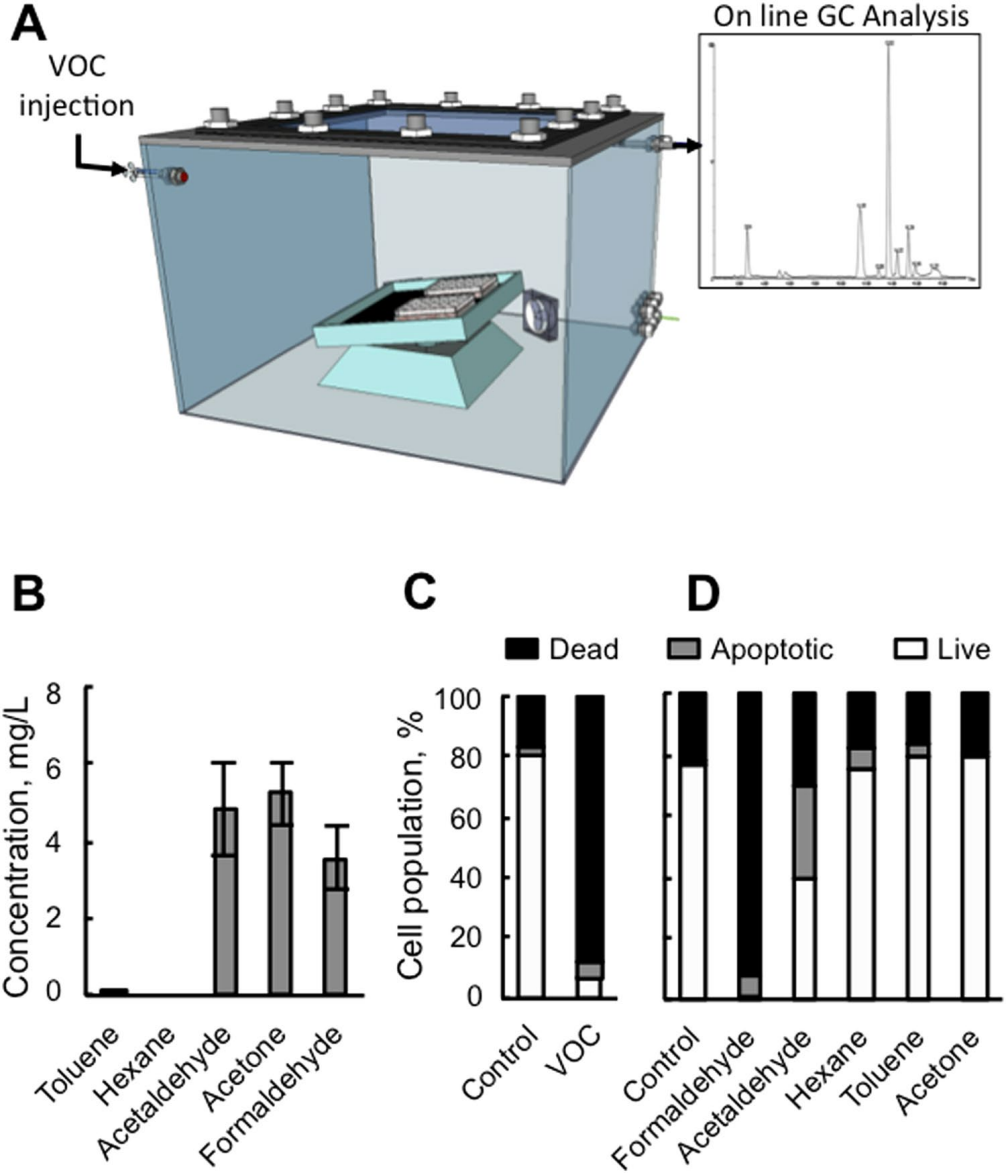
Effect of VOC exposure on keratinocytes viability. (**A**) VOC exposure system. The VOCs were injected through a septum and the VOC concentration ﻿in the gas phase was recorded by GC analysis and HPLC for formaldehyde. (**B**) Determination of VOCs in the medium (See Material and Methods). (**C**) Effect of combined VOC exposure on keratinocytes viability. Primary keratinocytes prepared from plastic surgery from a 30 year old healthy female donor were exposed to VOCs (toluene, hexane, acetaldehyde, formaldehyde, acetone, 20 ppmV, each) for 4 hours. Cells were stained with Annexin V-FITC and PI and analyzed by flow cytometry 24 hours after VOC exposure. Percentage of apoptotic cells (Annexin-PI positive) are shown by histogram. D. Effect of single VOC exposure. Keratinocytes were exposed to one VOC at a time, following the same experimental procedure. The data shown is representative of three separate cultures.

Primary keratinocytes were exposed to VOCs (toluene, hexane, acetaldehyde, formaldehyde and acetone, 0.8, 4 and 20 ppmV, each) for 4 hours in order to mimic real exposure to indoor gaseous pollutants (Fig. 1A and Fig. S1). We first quantified the concentration of each pollutant in cell medium in order to evaluate the VOC concentration the cell are really exposed to in the liquid (Fig. 1B). The concentrations of the pollutants inside the medium was measured by calibrated head space, solid phase microextraction gas chromatography–mass spectrometry (SPME GC-MS) analysis. Gas-liquid equilibrium between air and the medium could not be achieved under our experimental conditions (4 hours exposure, room temperature, small surface exchange) as shown by the differences between the very low concentrations in the medium and the theoretical concentration calculated from Henry’s law (see Materials and Methods and Table 1). Three VOCs were detectable in the cell medium, formaldehyde, acetaldehyde and acetone when using 20 ppmv of each VOCs for our study (Fig. S1). Hexane was not detectable in the liquid and is well known to damage cell membranes. A correlation between toluene and hexane hydrophobicity and induced toxicity has been shown by^11^. To determine whether these conditions lead to apoptosis, an Annexin/PI staining was performed 24 hours after VOC exposure. As shown in Fig. 1C, VOCs induced apoptosis in human primary keratinocytes. For apoptotic cells, a significant increase in late apoptosis (Annexin V+/PI+) was also observed (Fig. 1C). However, toluene, hexane or acetone alone did not induce apoptosis (Fig. 1D). On the contrary, exposure to 20 ppmv formaldehyde caused cellular death to the same extent as the VOC cocktail. Exposure to acetaldehyde alone resulted in 60% apoptosis. These results demonstrate that the most significant change in cellular viability is caused by formaldehyde and acetaldehyde and little from the mixture of the 5 VOCs.

**Table 1:** Dissolved VOCs measurement.

Molecule	Molar Mass (g mol^−1^)	Henry constant Pa m^3^ mol^−1^	*x*_*i*_ (ppmV)	Maximum concentration (mg L^−1^)	Measured concentration without cells (mg L^−1^)	Measured concentration with cells (mg L^−1^)
Acetaldehyde	44,05	8,89	20	9,7	4,84	3,33
Formaldehyde	30,03	2,65 10^−2^	20	2 200	3,55	5,12
Acetone	58,07	4,31	20	27,3	5,25	4,38
Toluene	92,14	6,73 10^2^	20	0,2	0,06	0,01
Hexane	86,17	1,7 10^5^	20	9,56 10^−4^	ND	ND

### VOC exposure induced alterations in mitochondrial transmembrane potential

**Figure 2:**
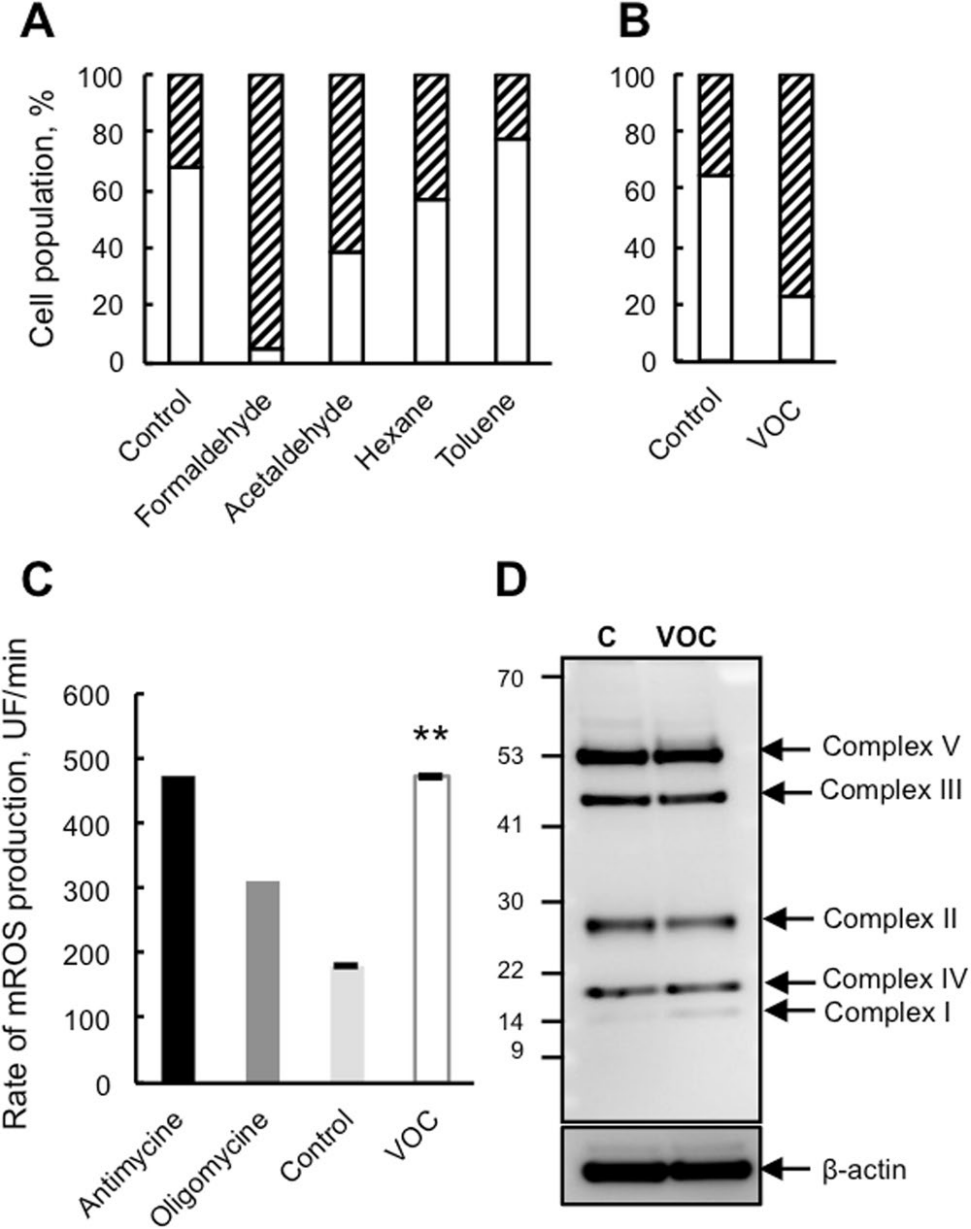
Collapse of the mitochondrial transmembrane potential and mitochondrial ROS production following VOC exposure. (**A**) Effect of single VOC exposure on mitochondrial potential. Primary keratinocytes were exposed to one VOC at a time (toluene, hexane, acetaldehyde, formaldehyde, acetone, 20 ppmV, each) for 4 hours. Mitochondrial membrane potential was measured using JC-1 by flow cytometry 24 hours post-treatment and expressed as a percent of cells with a normal membrane potential (white bars). The data shown is representative of three separate cultures. (**B**) Effect of combined VOC exposure on mitochondrial potential. (**C**) For the same VOC combined exposure as in panel A, the rate of superoxide production was measured in keratinocytes by flow cytometry using MitoSox. Antimycin (4 μg/ml) and oligomycin (1 μg/ml) induced a significant increase in mitochondrial ROS (mROS) production and were used as positive controls, **P < 0.01. (**D**) Immunoblot analysis of OXPHOS complexes (CI to CV) protein levels in cells following the combined VOC exposure, with actin as a loading control. The data shown is representative of three separate cultures. The blot was cropped and full-length blot is included in Supplementary file information.

Because mitochondria is implicated in apoptosis we sought further information regarding mitochondrial function. We subjected keratinocytes to the JC-1 cationic dye in a FACS analysis to examine whether the VOC exposure resulted in a drop in mitochondrial membrane potential (MMP). As shown in Fig. 2A, only exposure to formaldehyde and acetaldehyde alone demonstrated a significant decrease in JC-1 red-green fluorescence following treatment. In contrast, toluene and hexane treatment of the cells did not change their MMP. However, exposure to the VOC cocktail results in a drop in MMP similar to formaldehyde (Fig. 2B). To see whether this collapse could induce mitochondrial ROS production, we measured mitochondrial superoxide using Mitosox, a fluorogenic probe for the specific detection of superoxide in the mitochondria. VOC exposure induced an increase in mitochondrial ROS production (Fig. 2C). We used antimycin and oligomycin treated cells as controls, conditions known to induce mitochondrial ROS production. As shown in Fig. 2D, the steady-state amount of several components of the OXPHOS complexes, including subunits of the respiratory complexes I, II, III and IV, were not changed after VOC exposure.

### VOC compromised cellular antioxidant systems in cells and induced DNA damage

**Figure 3:**
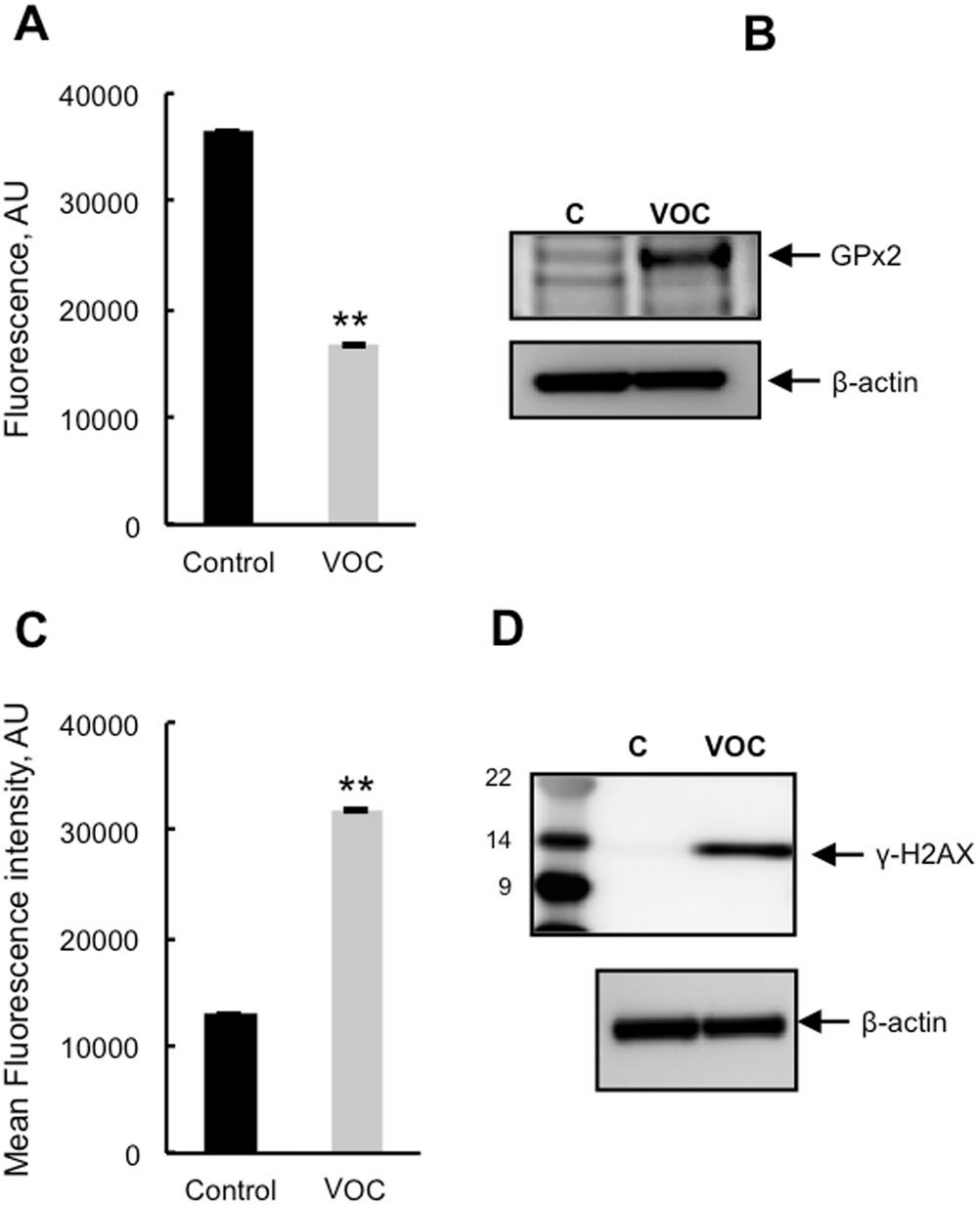
Antioxidant response and DNA damage following VOC exposure. (**A**) Measurement of reduced glutathione levels. Primary keratinocytes were exposed to VOCs (toluene, hexane, acetaldehyde, formaldehyde, acetone, 20 ppmV, each) for 4 hours. 24 hours after exposure they were incubated with ThiolTrackerTM Violet dye for 30 minutes and GSH level was assayed using a microplate fluorimetric reader. GSH level is presented as a percentage of non-treated cells. Data, mean ± SEM from three independent cultures, **P < 0.01 (**B**) GPx2 induction following VOC exposure. Primary keratinocytes were exposed to VOCs (toluene, hexane, acetaldehyde, formaldehyde, acetone, 20 ppmV, each) for 4 hours. 24 hours later GPx2 expression was detected by immunoblotting against GPx2 protein using cell lysates. The data shown is representative of three separate cultures. The blot was cropped and full-length blot is included in Supplementary file information. (**C**) For the same VOC exposure DNA damage was evaluated by flow cytometry using cells stained with Anti-phospho Histone H2A.X (Ser139) Alexa Fluor 488 antibody. Results are presented as the mean fluorescent intensity of the cells. Data, mean ± SEM from three independent cultures, **P < 0.01. For the same VOC exposure DNA damage was evaluated by western blot analysis using polyclonal antibody against Phospho-Histone H2AX. The blot was cropped and full-length blot is included in Supplementary file information.

Reduced glutathione (GSH) is the first scavenger of the cell by its conversion to oxidized glutathione (GSSG). To evaluate cellular antioxidant response to VOC treatment we measured GSH. As shown in Fig. 3A, VOC treatment induced a distinct decrease in GSH levels in keratinocytes. This drop in glutathione was concomitant with glutathione peroxidase 2 induction which is the first H_2_O_2_ degrading enzyme in the cell (Fig. 3B). To determine if VOC exposure could induce DNA damage, we looked at phosphorylation of H2AX which is used to quantify accumulation of DNA damage. Western blot against histone H2AX phosphorylated at Ser-139 revealed that VOCs induced an increase in DNA damage in keratinocytes (Fig. 3C and D).

### VOC exposure induced lipid peroxidation and oxidative damage to proteins

**Figure 4:**
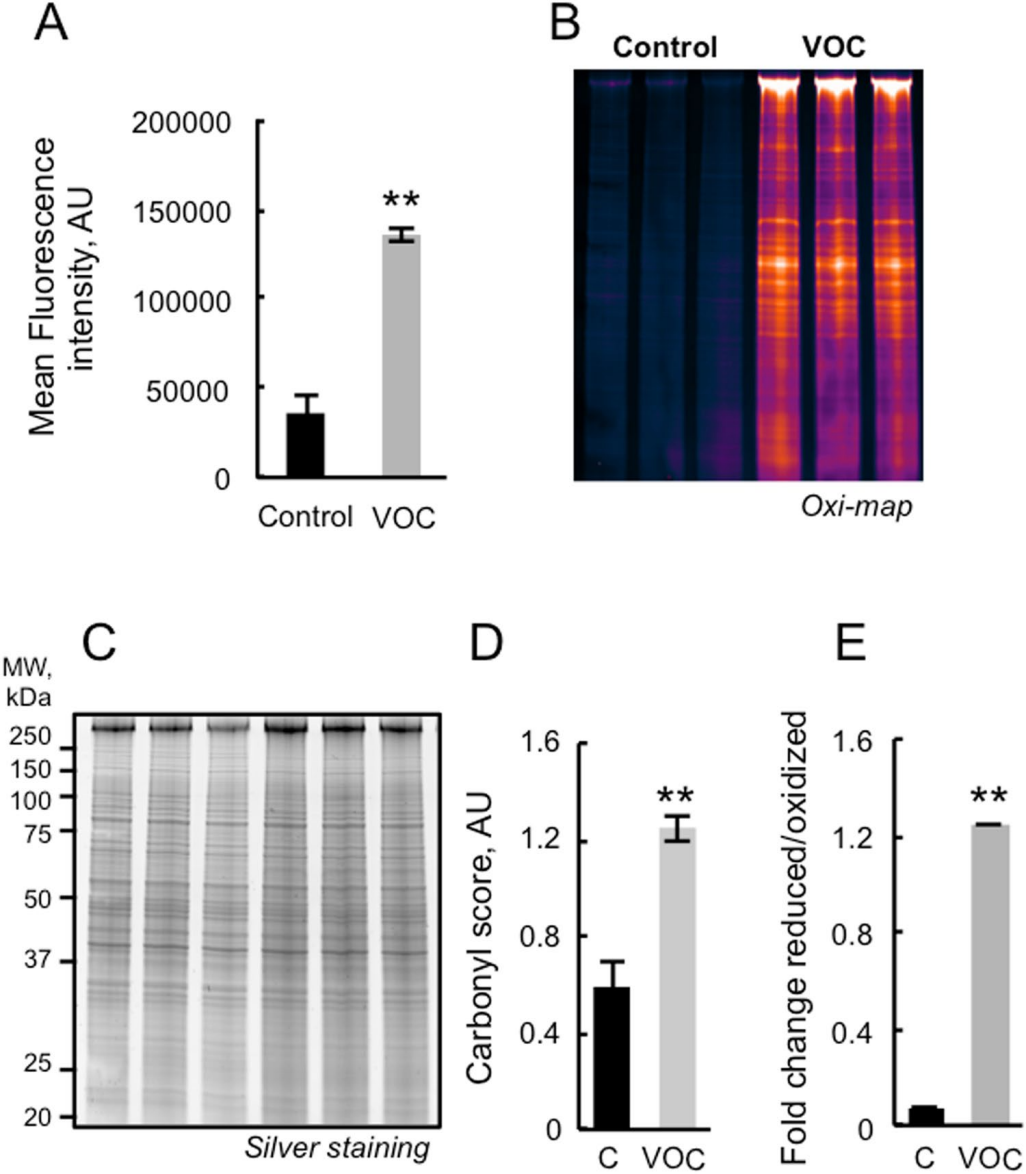
Detection of oxidatively modified proteins and lipid peroxidation following VOC exposure. (**A**) Primary keratinocytes were exposed to VOCs (toluene, hexane, acetaldehyde, formaldehyde, acetone, 20 ppmV, each) for 4 hours. After 24 hours, carbonylated protein within cells was evaluated by flow cytometry using cells stained with Anti-FITC conjugated monoclonal antibody that specifically binds to DNP moiety. Results are presented as the mean fluorescent intensity of the cells. Data, mean ± SEM from three independent cultures,** P < 0.01. (**B**) Fluorescent detection of oxidized proteins. Keratinocytes were exposed to the same cocktail of VOCs. After 24 hours, cells were analyzed by SDS-PAGE (4–20%) pattern of carbonylated proteins pre-labeled with C5Hz (Cy5 hydrazine labeling). The gel was cropped and full-length gel is included in Supplementary file information. (**C**) Total proteins post-stained with Protein GOLD^TM^. The gel was cropped and full-length gel is included in Supplementary file information. (**D**) Semi-quantification of carbonylated proteins were performed by densitometric analysis, expressed as relative values and shown as mean ± S.D (n = 3) and analyzed using Student’s t-test; **P < 0.01. (**E**) Lipid peroxidation detection with Image-iT Peroxidation Kit. Visualization of lipid peroxidation *in-situ* was carried out through labeling cells with C11-BODIPY581/591, a fatty acid analogue that readily incorporates into cell membranes and whose fluorescence irreversibly changes from red to green upon exposure to ROS. Keratinocytes were stained with 10 µM C11-BODIPY581/591for 30 minutes, exposed to VOCs and analyzed after treatment with a microplate fluorimetric reader. In control cells, most of the signal is in the red channel and the ratio of 590/510 (reduced/oxidized) is high. Data, mean ± SEM from three independent cultures, **P < 0.01.

VOC exposure results in increased rates of free radical production by the mitochondria. Thus, using immunological detection of protein carbonyls, we sought information on relative alterations in the levels of oxidized proteins due to pollutant treatment. Carbonyl functional groups can be introduced into proteins by a variety of oxidative processes including direct oxidation of amino acid by ROS or reaction of lipid peroxidation products from cellular membrane oxidation. As shown in Fig. 4A, VOC treatment of keratinocytes induced a distinct increase in the levels of oxidatively modified protein in cells. In order to be more sensitive and to quantify the level of protein carbonylation we used a fluorescent Oxi-map method. As shown in Fig. 4B oxidative modification was global in nature. We also measured total lipid peroxidation production in cells, as shown in Fig. 4E, we detected an increase in lipid peroxidation after exposure of the cell to VOCs.

### VOC exposure induced alterations in proteasome and Lon protease peptidase activity in keratinocytes

**Figure 5:**
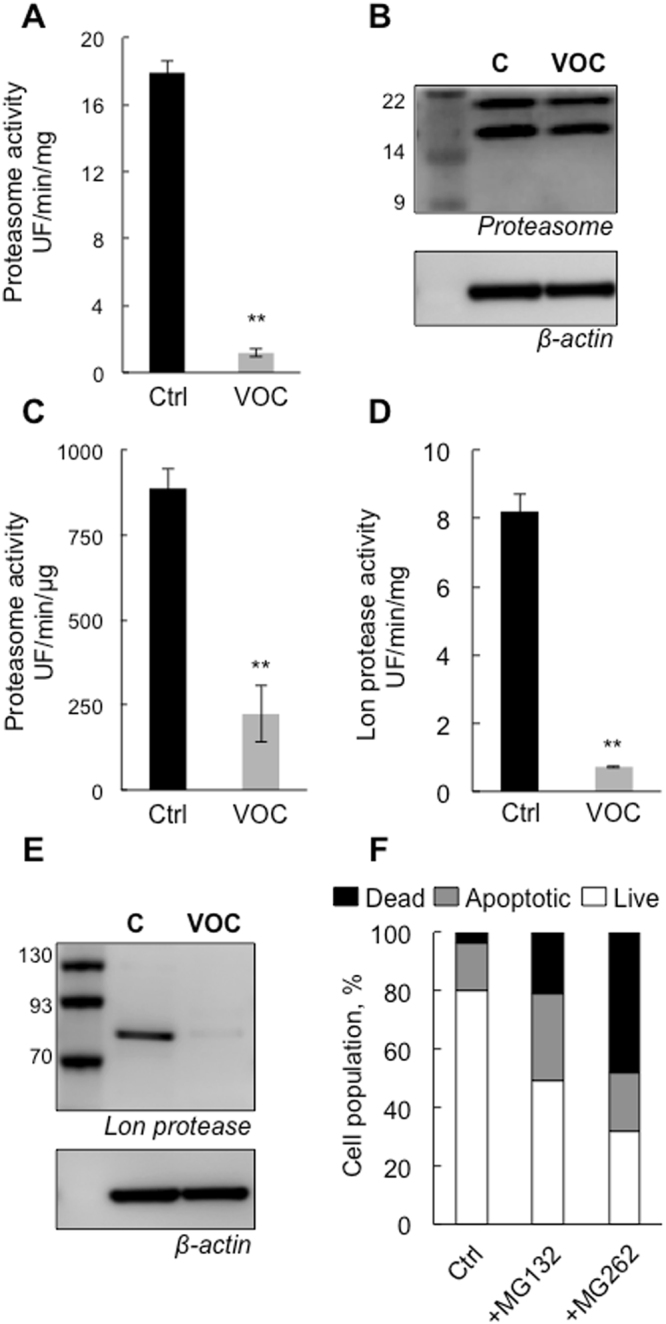
Proteasome and Lon protease inactivation following VOC exposure. (**A**) Primary keratinocytes were exposed to VOCs (toluene, hexane, acetaldehyde, formaldehyde, acetone, 20 ppmV, each) for 4 hours. After 24 hours, proteasome chymotrypsin-like activity was measured 24 hours post-treatment using the fluorogenic peptide LLVY-AMC. Proteasome activity is presented as a percentage of non-treated cells. Data, mean ± SEM from three independent cultures **P < 0.01. (**B**) Proteasome expression was evaluated by western blot analysis using polyclonal antibody against 20 S proteasome, whole-cell lysates, the data shown is representative of three separate cultures. The blot was cropped and full-length blot is included in Supplementary file information. (**C**) Effect of VOC on proteasome chymotrypsin-like activity *in vitro*. 20 S proteasome purified from rabbit (2 μg of purified enzyme per assay) was incubated for 30 min with with 4.84 mg L^−1^ acetaldehyde, 3.55 mg L^−1^ formaldehyde, 5.25 mg L^−1^ acetone and 0.06 mg L^−1^ toluene at room temperature. These concentrations are the measured in the liquid media of the cells (Table 1). Proteasome chymotrypsin-like activity was measured post-treatment using the fluorogenic peptide LLVY-AMC. Data, mean ± SEM from three independent experiments **P < 0.01. (**D**) For the same VOC treatment as in panel A, mitochondria were isolated and matrix were prepared. Lon protease activity was determined using 5 μg of purified matrix and 100 μM of FRETN 89-98 in the presence of 1 mM ATP. Data, mean ± SEM from three independent cultures, **P < 0.01. (**E**) Lon protease expression was evaluated by western blot analysis using polyclonal antibody against human Lon. The data shown is representative of three separate cultures. The blot was cropped and full-length blot is included in Supplementary file information. (**F**) Primary keratinocytes were treated with either 10 μM Lon inhibitor MG262 or 10 μM proteasome inhibitor MG 132 for one hour and exposed to VOCs (toluene, hexane, acetaldehyde, formaldehyde, acetone, 20 ppmV, each for 1 hour). Cells were stained with Annexin V-FITC and PI and analyzed by flow cytometry 24 hours after VOC exposure.

The ubiquitin-proteasome system is an important regulator of cell growth and apoptosis^13^. VOCs produce ROS that lead to the production of oxidized proteins which are preferentially degraded by the proteasome (Fig. 4). Thus the effects of VOC exposure on proteasome activity were evaluated 24 hours later. As shown in Fig. 5A proteasome was inactivated in keratinocytes. The decline in proteasome activity was not due to a loss of proteasome content as judged by Western-blot analysis in Fig. 5B. To determine whether the observed decline of proteasome activity could be explained by direct inactivation of the 20 S proteasome by VOCs, chymotrypsin –like activity was assayed after incubation of purified proteasome with concentrations of these compounds measured in the liquid media of the cells (Table 1). The results presented in Fig. 5C indicate that VOCs can inactivate proteasome activity. This finding raises the possibility that the proteasome may be directly oxidized by the VOCs such as formaldehyde but an inhibitory effect of oxidized protein cannot be excluded. Mitochondria possess a number of proteases that degrade misfolded and oxidatively modified proteins^14^. In order to investigate if the accumulation of oxidized proteins in keratinocytes exposed to VOCs was due to a diminished capacity for removal of protein in the mitochondria, we measured Lon protease activity. To specifically monitor its activity, we isolated mitochondrial matrix and used a peptide reporter, FRETN89-98, which has been shown to be degraded by human Lon but not by other mitochondrial matrix proteins such as Clp^15^. As shown in Fig. 5D, a decline in Lon protease activity was observed. We next investigated the possible regulation of Lon protease at the protein level. It has recently been shown that Lon induction and *de novo* synthesis during oxidative stress adaptation is protective against the accumulation of oxidative protein damage^16^. Western blot analysis revealed a significant decline in Lon expression after VOC treatment suggesting that cells did not cope with VOC exposure by inducing Lon (Fig. 5E). It is well known that proteasome dysfunction is a consequence of oxidative stress and that proteasome inhibition induces mitochondrial dysfunction. Treatment of cells with MG132, a specific inhibitor of the proteasome prior exposure to VOCs treatment for one hour induces apoptosis (Fig. 5F). In contrast, MG262, an inhibitor of Lon protease had stronger effect on cell viability suggesting that Lon inhibition would impair cellular viability toward VOCs exposure. We cannot expose the cells for more than one hour to VOCs because it was very toxic, that is why the level of cell apoptosis are not the same that the on obtained in Fig. 1C.

### VOC exposure induced decline in cell proliferation and alterations in proteasome activity in cutaneous tissue

**Figure 6:**
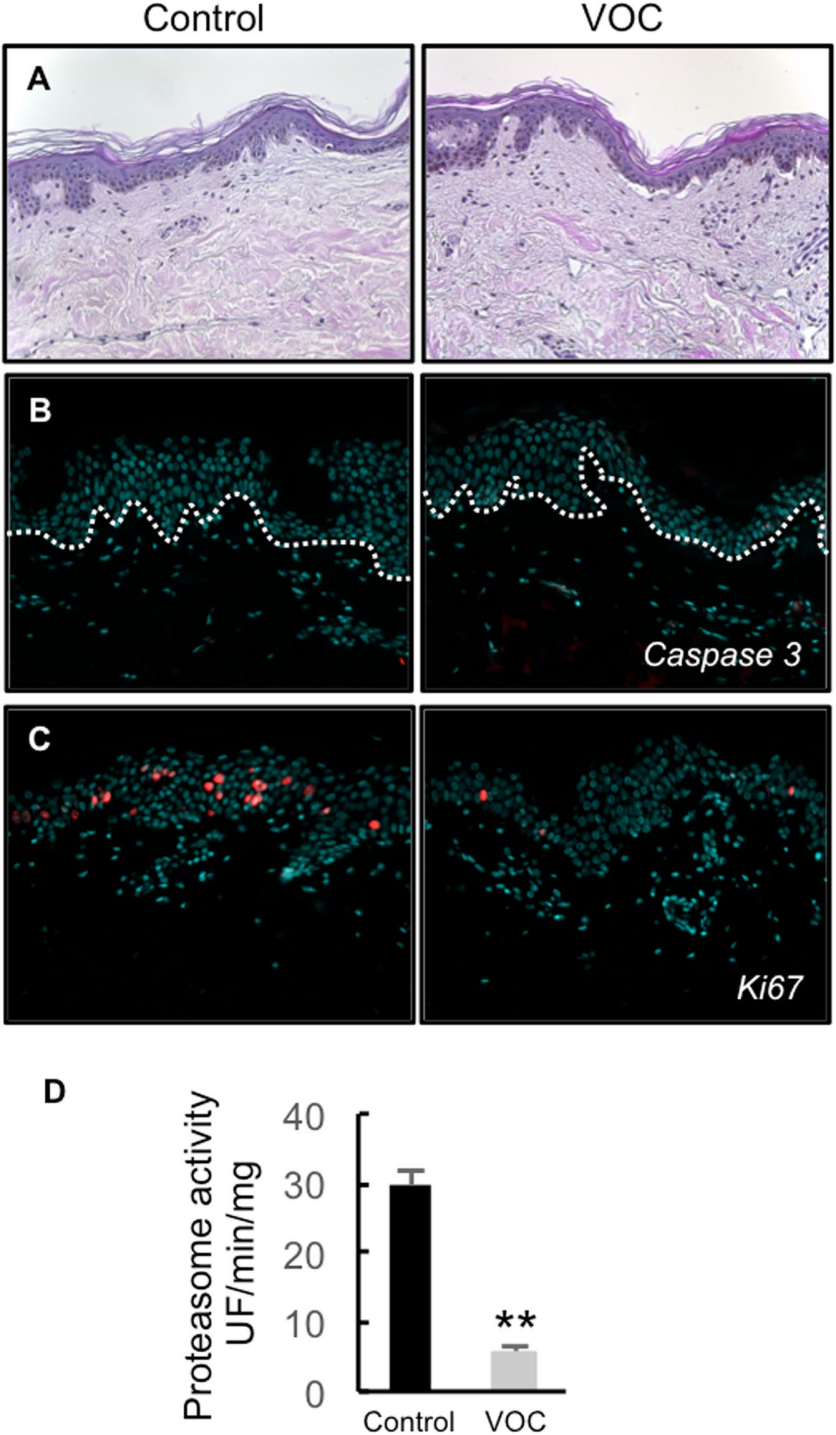
Effect of VOC exposure on skin explants. (**A**) Hematoxylin and eosin staining. NativeSkin models from a 30 year old donor female were cultured for 4 days with NativeSkin culture medium, exposed or not to VOCs (toluene, hexane, acetaldehyde, formaldehyde, acetone, 80 ppmV, each) for 4 hours every day. 24 hours after the last VOC exposure, samples were fixed in formalin and embedded in paraffin wax. 5 µm skin cross-sections were stained with hematoxylin eosin. Scale bar is 100 µm. (**B**) Apoptosis analysis. 5 µm skin cross-sections were immuno-stained for active caspase 3 to detect apoptotic cells. All pictures are representative of the whole sample. Scale bar is 100 µm. (**C**) Cell proliferation assessment. 5 µm skin cross-sections were immuno-stained for Ki-67. All pictures are representative of the whole sample. Scale bar is 100 µm. (**D**) proteasome activity. 24 hours after the last VOC exposure the epidermis was separated from the dermis and homogenized with a Dounce homogenizer and proteasome chymotrypsin-like activity was measured 24 hours post-treatment using the fluorogenic peptide LLVY-AMC. Data, mean ± SEM from 4 skin explants from two different donors (30, and 33 year old) females were used. **P < 0.01.

Four skin explants from two different donors were used in this study (30, and 33 year old) females to test any difference in sensitivity to VOCs between individuals. Skin explants (NativeSkin models) were exposed to the VOC cocktail (toluene, hexane, acetaldehyde, formaldehyde and acetone, 80 ppmV, each) for 4 hours, every day for 4 days. 24 hours after the last exposure, we first analyzed the cell viability in the treated skin samples. We found a significant decrease in cell viability in the NativeSkin models exposed to pollution compared to untreated controls (Fig. S2C). We also analyzed tissue integrity in treated and untreated NativeSkin models (Figs 6A and S2A). Untreated controls presented with normal tissue integrity up to day 4 of *ex vivo* culture. In contrast, we found slight defects underlying cell death in the NativeSkin models exposed to pollution. The anomalies observed included spongiosis, pyknotic nuclei, and even epidermal detachment (Figs 6A and Fig. S2A). To quantify the skin defects observed, we established a simple histopathological scoring for each skin sample (Fig. S2D), based on the cumulative counting of the cell anomalies mentioned above (spongiosis, pyknotic nuclei and epidermal detachment). To better understand the exact nature of the cell death (apoptosis or necrosis) observed in the NativeSkin models exposed to pollution, we investigated the expression of activated caspase 3 by immunofluorescence. Surprisingly, we did not detect any sign of apoptosis in both untreated and treated NativeSkin models (Fig. 6B). This suggested that the exposure to pollution induced cell necrosis in the NativeSkin models and not apoptosis. We finally analyzed cell proliferation by staining Ki-67 in NativeSkin models. We observed that proliferative cells were normally present in the non-polluted NativeSkin models from day 0 to day 4 of *ex vivo* culture (Fig. 6C and Fig. S2A). Interestingly, exposure to pollution induced a marked decrease of Ki-67 positive cells in the epidermis for both treated and untreated NativeSkin models. Because proteasome is a key enzyme in the cell cycle regulation we also measured its activity in NativeSkin models. Exposure to VOCs resulted in a huge decline in proteasome activity (Fig. 6D).

In conclusion, we demonstrated in this study that exposure to pollution decreased cell viability and proteasome activity in the *ex vivo* human skin model NativeSkin. We did not detect any difference in the sensitivity to VOCs exposure between individuals. These anomalies are confirmed at the histological level, by the presence of cell necrosis features including spongiosis, pyknotic nuclei and dermoepidermal detachment. Exposure to pollution also led to a reduction in total cell RNA and nuclear DNA content, as shown by methyl green and pyronin Y staining (data not shown). Interestingly, no marked sign of apoptosis were found in the NativeSkin samples exposed to pollution.

## Discussion

This study evaluated the effects of VOC exposure on keratinocytes. This is the first study directly exposing human skin explants to such a cocktail. *In vitro* models are a promising approach to characterizing the impact of indoor air pollution on skin. Generation of pollutants in the system was achieved at concentrations that are representative of indoor pollution and the real time monitoring in the system allows us to ensure that their concentrations are stable during the whole cell exposure^5, 6, 17, 18^. Unlike the cells, the biopsies were in direct contact with polluted air. Except with formaldehyde, these concentrations were in agreement with limit exposure values given by INERIS and WHO^6^. Formaldehyde is the only product with data on its cutaneous toxicity. *In-vivo* and epidemiological studies were performed with high concentrations (0.1 to 1%) of formaldehyde in direct contact with the skin^19, 20^. So far, repeated toxicity studies on VOCs have been carried out within the context of animal studies only^8, 21, 22^. Consequently, we had to adapt the doses of VOC exposure to mimic human exposure to indoor pollutants.

Interaction between VOCs and cell membranes play a key role in VOC-induced toxicity by inducing ROS production. This ROS production can damage proteins, DNA of the cells but also can trigger inflammation by activation of cytokines and might be involved in the initiation or pathogenesis of allergic or non-allergic cutaneous inflammation^23^. Genotoxic effects have repeatedly been observed in cultured human cells *in-vitro* ^24^. It is well known that the primary DNA alternations after formaldehyde exposure are DNA–protein cross-links (DPCs)^25^. The DPCs can arrest DNA replication and lead to the induction of other genotoxic effects such as sister chromatid exchanges (SCE) and micronuclei (MN) in proliferating cells. We have shown that exposure to VOCs lead to DNA damage that may be explained mainly by the formaldehyde (Figs 1D, 3C,D). Although little is known of the toxic mechanism of formaldehyde, Teng *et al*., (2001) have reported formaldehyde-induced ROS production^26^. Various types of protein oxidative modifications are directly or indirectly induced by ROS by reactions with secondary products of oxidative stress^27^. Cysteine and methionine residues are particularly prone to oxidative modifications, but they might not be directly linked to protein damage, since they also participate in cellular signaling events^28^. On the other hand, irreversible oxidation products of other residues are most frequently hydroxylated and carbonylated amino acid side chain derivatives. We found that exposure to VOCs results in the build-up of oxidized proteins (Fig. 4A–D) and proteasome inhibition. (Fig. 5A,C). Thus, the inability to efficiently remove modified protein may impact a variety of cellular pathways.

We found that exposure to VOCs resulted in an increase of lipid peroxidation (Fig. 4E). 4-hydroxy-2-nonenal (HNE) is an indirect mediator of oxidative stress, formed by lipid peroxidation of polyunsaturated fatty acids in, for example, cholesterol, phospholipids and triglycerides^29^. HNE is highly reactive and able to readily modify the sulfhydryl group of cysteine residues and the imidazole group of histidine residues, while the ε-amino group of lysine residues serves as a less active target for nucleophilic attack by HNE. Hence unstable HNE-adducts could be overlooked as a regulatory mechanism of proteasomal activity and a participating factor in the decreased proteasomal activity associated with oxidative stress. We have shown that proteasome activity was inhibited upon VOC exposure but not its expression suggesting that the proteasome may be a target of HNE (Fig. 5A–C)^30, 31^. Formation of inhibitory proteins is an additional mechanism by which free radicals may affect proteasome function upon VOC exposure. We and others have previously shown that proteins cross-linked by HNE are resistant and can act as a potential inhibitor of proteasome^30, 32^. Our results suggest that ROS and lipid peroxidation products may react directly with proteasome (Fig. 5C) or with other proteins that may act as proteasome inhibitors. Ongoing studies seek to identify specific sites on the proteasome subunits which have carbonylated proteins, as we have shown by Oxi-map (Fig. 4B), these may act as substrate or inhibitor of the enzyme.

ROS induced -VOC target cells via mitochondrial dysfunction and activation of oxidative stress signaling pathways. We have recently shown that a defect in Lon protease resulted in proteasome inhibition in yeast^33^. It is well known that proteasome dysfunction is a consequence of oxidative stress and that proteasome inhibition induces mitochondrial dysfunction^34^. There is a close association between mitochondrial electron transport activity and the regulatory caps of the 26 S proteasome, which are ATPases. This creates a complex picture, in which several pathways are interconnected. Our hypothesis, which needs to be confirmed, is that the primary event is mitochondrial ROS production in cells exposed to VOCs, maybe because of increased leaks in the respiratory chain resulting in Lon protease inhibition (Figs 2 and 5F).

We have previously shown that UV-irradiation of keratinocytes induces a significant decline in proteasome function and is correlated with an increase in oxidatively modified and ubiquitinated proteins and participate in photoaging^35^. Evidence from several lines of investigation has indicated that photoaging, when superimposed on the intrinsic aging process, plays a major role in age-associated degenerative changes of the skin^36^. There is also evidence that these processes, at least in part, overlap, since they both have a free radical component^36^.In addition, our results suggest that exposure to pollutants which also target proteasome function may play a role in accelerating skin aging. As already stated above, a hallmark of cellular aging is the accumulation of oxidatively damaged proteins that have been associated with both increased ROS production and alteration of protein maintenance systems such as the proteasome.

In summary, our results provide support for the hypothesis that VOC treatment induced free radical production that mediate apoptosis due to DNA damage, mitochondrial potential collapse, lipid peroxidation and proteasome inactivation in keratinocytes cells and in human skin explants. Ongoing efforts to identify species oxidized protein that are not efficiency degraded following VOC exposure will enable elucidation of the long-term consequences of proteasome inhibition on specific cellular and cutaneous function.

## Methods

### Chemicals

All chemicals were purchased from Sigma-Aldrich (Saint Quentin Falavier, France).

### Ethic statements

With respect to the ethical permissions, for studies using skin biopsies for cell culture experiments, the principle requirements of the Declaration of Helsinki were taken into account to protect the rights, safety and well-being of subjects participating in the study. Before initiating the studies, the investigator had obtained written consent from the participants and full approval from the Freiburg Ethics Commission International for the protocol, protocol amendment(s), if applicable. All participants who provided their skin biopsies for this research provided their written informed consent to participate in this study and for their data to be used for research purposes. For cell culture studies, skin samples were collected from adult patients, undergoing plastic surgery performed by independent plastic surgeons and were considered as “waste”, and thus were exempt from any further approval. Even in this case, verbal informed consent from the participants that provided their waste tissues for research purposes was obtained. All the data in this study was anonymized. All the data in this study was anonymized (all relevant documents available at LVMH-Research, St. Jean de Braye, France).

NativeSkin models were produced from anonymized skin samples collected from the plastic surgery department of Toulouse Hospital. With respect to the ethical permission, for studies using human biological material, the principle requirements of the Declaration of Helsinki were taken into account to protect the rights, safety and well-being of subjects participating in the study. Genoskin holds a permit (AC-2011-1443) granted by the French Ministry of Higher Education and Research to collect, transform, commercialize and export human biological material for scientific research use. Before initiating the studies, the investigator had obtained written consent from the participants and full approval from the French ethical committee CPP (Comité de Protection des Personnes), to participate in this study and for their data to be used for research purposes.

### Cell culture

Primary cultures of human dermal keratinocytes were prepared from skin biopsies from LVMH-Research, St. Jean de Braye, France and were grown in Epilife medium (Thermofischer, Saint Aubin, France). Skin samples were collected from a 30 year old healthy female donor patient, undergoing plastic surgery (mammary gland reduction) performed by independent plastic surgeons and were considered as “waste”. Even in this case, verbal informed consent from the participants that provided their waste tissues for research purposes was obtained. All the data in this study was anonymized (all relevant documents available at LVMH-Research, St. Jean de Braye, France). The Lon protease and proteasome inhibitors used were 10 μM MG262 (Boston Biochem) and 10 μM MG132 (Sigma-Aldrich).

### VOC exposure

VOC exposure was carried out in a 144 L tight box equipped with two homogenization fans, a swirling table, a temperature and hygrometry sensor and an upper glass window. Before each experiment, the box was flushed with water-saturated clean air (%RH > 80% at 21 °C). The culture plates containing cells or biopsies were placed on the swirling table (the plate’s covers were left in place). Then after tightly closing the box, the VOC solutions were injected through a septum, homogenization was obtained in few seconds with the two fans. The VOC concentration in the gas phase was recorded by automatic sampling with a GC-FID Varian 3800 equipped with a 250 μl sampling loop. Formaldehyde was quantified at the end of experiment by collecting 1 liter of air on an LpDNPH S10 Cartridge (Supelco) and HPLC analysis was carried out according to the manufacturer’s recommendations. Keratinocytes were exposed to 0.8, 4 or 20 ppmV of each VOC (formaldehyde, toluene, acetaldehyde, acetone and hexane) for 4 hours.

### Dissolved VOC measurement

The dissolved VOC in the medium was quantified with head-space SPME GC-MS analysis^16^. For each compound, 2 mL of exposed medium were placed in a 10 mL flask and heated for 5 minutes at 30 °C. PBFBHA impregnated SPME fibers for the sampling of aldehydes and PDMS/Carboxen SPME fibers for acetone, hexane and toluene were exposed for calibrated times^37^. Concentrations were expressed in mg L^−1^ of medium.

### Calculation of the maximum concentration in the culture medium

According to the Henry’s law, the maximum concentration of each VOC in the medium was determined assuming that the medium may be considered as water. Our experimental results were always lower than these calculated values, implying that the gas-liquid equilibrium was not reached after 4 hours of exposure. Formaldehyde concentration is noticeably lower in the cell culture medium than the other polar pollutants, but its well-known high reactivity with the polar molecules of the media could explain these quite low concentrations.

1 $${K}_{H}^{pc}=\frac{{P}_{i}}{Conc}$$

Équation 1: Henry’s law volatility constant ${{\boldsymbol{K}}}_{{\boldsymbol{H}}}^{{\boldsymbol{pc}}}$ (Pa m^3^ mol^−1^) is defined by the ratio between the partial pressure (*P*_*i*_ = *x*_*i*_ *P*_*atm*_) of the component in gas phase and its concentration in water phase (mol m^−3^).

### Flow cytometry analysis of apoptosis

An Annexin V-FITC/PI apoptosis detection kit was used as described by the manufacturer (Thermofischer, Saint Aubin, France). Flow cytometric analysis of apoptotic populations, was carried out using a BD Accuri™ C6 flow cytometer (BD Biosciences, Le Pont de Claix, France).

### Flow cytometry analysis of DNA damage

A FlowCellect™ Cell Cycle Checkpoint H2A.X DNA Damage detection kit was used as described by the manufacturer (Millipore, Molsheim, France). Flow cytometric analysis of d cells stained with Anti-phospho-Histone H2A.X (Ser139) Alexa Fluor 488 antibody was carried out using a BD Accuri™ C6 flow cytometer (BD Biosciences, Le Pont de Claix, France).

### Lipid peroxidation analysis

Visualization of lipid peroxidation *in-situ* was carried out through labeling cells with C11-BODIPY581/591, a fatty acid analogue that readily incorporates into cell membranes and whose fluorescence irreversibly changes from red to green upon exposure to ROS (Image it, Thermofischer, Saint Aubin, France). Samples were then analyzed at 37 °C using a microplate fluorimetric reader (BMG-FLUOstar Galaxy, Stuttgart, Germany). Red emission from intact C11-BODIPY581/591 was detected at 580–620 nm and green emission that indicated peroxidation at 495–560 nm.

### Determination of cytosolic glutathione levels (GSH)

Glutathione levels were determined using Thiol tracker violet detection reagent (Thermofischer, Saint Aubin, France) and analyzed using a microplate fluorimetric reader (BMG-FLUOstar Galaxy, Stuttgart, Germany), excitation/emission wavelengths were 405/526 nm.

### Mitochondrial membrane potential

Mitochondrial inner membrane potential was measured as previously described^38^ using JC-1 probe (Thermofischer, Saint Aubin, France).

### Mitochondrial ROS production

Cells were trypsinized and counted using a BD Accuri™ C6 flow cytometer (BD Biosciences, Le Pont de Claix, France). Cells were incubated with a 5 μM MitoSOX™ probe (Thermofischer, Saint Aubin, France) for 5 minutes at room temperature, and incubated with different media: standard medium, with oligomycin (2 μg/ml) or with antimycin (4 μg/ml). Fluorescent kinetics were recorded in the flow cytometer for 10 minutes.

### Proteasome peptidase activity

Peptidase activity of the proteasome was assayed using a fluorogenic peptide, succinyl-Leu-Leu-Val-Tyr-7-Amido-4-Methylcoumarin (LLVY-AMC) Sigma-Aldrich (Saint Quentin Falavier, France) as previously described^30^.

For inactivation experiments, 2 μg of purified proteasome (20 S Proteasome fraction from rabbit, Saint Quentin Falavier, France) were incubated with 4.84 mg L^−1^ acetaldehyde, 3.55 mg L^−1^ formaldehyde, 5.25 mg L^−1^ acetone and 0.06 mg L^−1^ toluene at room temperature for 30 min. After preincubation, proteasome peptidases activities were determined as described above.

### Monitoring specific activity of Lon protease from isolated keratinocyte mitochondrial matrix

Mitochondria were isolated by differential centrifugation, as previously described^38^. Matrix preparation was carried out as previously described^39^, by hypotonic shock followed by sonication (1 minute, 3 times) in 10 mM HEPES buffer (without EDTA; pH 7.2) and centrifugation (100,000 g, 60 minutes, 4 °C). Methods and materials for the synthesis of peptide substrate FRETN 89–98 and inhibitor DBN9310 are detailed in ref. 15. Reactions containing 50 mM HEPES (pH 8), 5 mM Mg(OAc)2, 2 mM DTT, 5 mM imidazole, and 100 µM FRETN 89–98 in the absence and presence of 1 mM ATP were incubated at 37 °C for 1 minute. Five micrograms of the purified matrix from keratinocytes mitochondria culture were added and peptide cleavage was observed by monitoring the fluorescent emission at 420 nm (λ_ex_ = 320 nm) for 1 hour.

### Western blot analysis

Cellular lysis was performed using a lysis buffer (1.5 mmol/L EDTA, 50 mmol/L Hepes pH 7.4, 150 mmol/L NaCl, 10% (v/v) glycerol, and 1% (v/v) NP40). Total cellular lysates were loaded onto a 4% to 20% SDS-PAGE gel (Bio-Rad), transferred onto nitrocellulose membrane, and revealed with different antibodies such as homemade anti-proteasome^40^ and anti-Human Lon protease^41^. Commercial antibodies used were, MitoProfile Total OxPhOS WB Antibody Cocktail and anti-phospho-gamma H2AX-Ser139 (Thermofischer, Saint Aubin, France), anti-actin and anti-GPx2 (Abcam, Paris, France). Direct recording of the chemi-luminescence (GeneGnome Syngene) and quantification (GeneSnap software) were performed (Ozyme, St Quentin en Yvelines, France).

### Detection of carbonylated proteins

Carbonylated proteins in cells were detected and analyzed after the derivatization of protein carbonyl groups with 2,4-dinitrophenylhydrazine (DNPH) and the detection of DNP-derivatized proteins with a FITC conjugated monoclonal antibody that specifically binds to DNP moiety (Flowcellect^TM^ oxidative stress characterization kit, Millipore, Molsheim, France). Flow cytometric analysis allows the quantification of oxidized protein within the cells. For oxi-map analysis (Oxiproteomics, Paris, France), carbonylated proteins were labeled with CyDyeTM hydrazides (GE Healthcare) as previously described^42, 43^. Carbonylated proteins were labeled with Cy5 hydrazides (GE Healthcare) and total proteins were precipitated and resuspended in loading buffer and separated by SDS-PAGE (4–20%). Total proteins were post-stained with ProteinGOLD (Gel Company). Fluorescent scanning was performed using the Ettan Dalt system (GE Healthcare) at excitation and emission wavelengths of 635/680 nm for the C5Hz (Cy5 hydrazide) and 390/595 nm for total proteins, respectively. Semi-quantification of carbonylated proteins were performed on digitalized images by densitometric analysis.

### NativeSkin production and treatments

Skin explants, NativeSkin models were purchased at Genoskin (Toulouse, France). NativeSkin models were produced from anonymized skin samples collected from the plastic surgery department of Toulouse Hospital. 4 skin explants from two different donors were used in this study (30, and 33 year old) females presenting no dermatological disease. With respect to the ethical permission, for studies using human biological material, the principle requirements of the Declaration of Helsinki were taken into account to protect the rights, safety and well-being of subjects participating in the study. Genoskin holds a permit (AC-2011-1443) granted by the French Ministry of Higher Education and Research to collect, transform, commercialize and export human biological material for scientific research use. Before initiating the studies, the investigator had obtained written consent from the participants and full approval from the French ethical committee CPP (Comité de Protection des Personnes), to participate in this study and for their data to be used for research purposes. NativeSkin models were cultured in a CO_2_ incubator at 37 °C for 4 days. For VOC exposure, NativeSkin models were incubated in the chamber for 4 hours each day for 4 days and exposed to 80 ppmV of each VOC (formaldehyde, toluene, acetaldehyde, acetone, hexane). The medium was replaced after VOC exposure.

### MTT tests of NativeSkin models

One quarter of NativeSkin samples were immersed in 500 µL of 0.5 mg/mL MTT in PBS then incubated for 17 hours at 37 °C. Resulting formazan was extracted in 500 µL isopropanol 6 h at RT under shaking. Optical density was read for 200 µL at 570 nm.

### Proteasome analysis of NativeSkin models

Skin samples were incubated for 5 minutes at 56 °C in 10 mM EDTA. The epidermis was separated from the dermis and homogenized with a Dounce homogenizer in extraction buffer (40 mM Tris pH 7.5, 1 mM EDTA, 5 mM 2-mercaptoethanol and 20% (v/v) glycerol). Membranes and cellular debris were eliminated by centrifugation at 10,000 g and the soluble extract was recovered. Protein concentrations were determined by BioRad-protein-assay (Bio-Rad, Ivry, France). Peptidase activities of the proteasome were assayed by using fluorogenic peptide LLVY-AMC for the chymotrypsin-like.

### Immunohistochemistry of NativeSkin models

Formalin-fixed skin samples were incubated in 70° ethanol. Samples were dehydrated then paraffin embedded. Samples were cut in 5 µm sections for staining or immunofluorescence. Hematoxylin eosin: Sections were deparaffinized then immersed in Mayer’s hematoxylin during 3 minutes, rinsed in water, transferred in 95° ethanol, immersed in eosin for 2 minutes, rinsed in 95° ethanol then dehydrated for mounting. For immunofluroescence, sections were deparaffinized then rehydrated in PBS. Antigen retrieval was performed for 45 minutes at 95 °C. Sections were then saturated with a 1/20 goat serum in PBS, for 45 minutes at 37 °C. Sections were then covered with primary antibody (anti caspase-3, R&D systems AF835; anti Ki-67, Dako, M724029) diluted in 1/20 goat serum in PBS, and stored overnight at 4 °C. After 2 washes in PBS, section were covered with the secondary antibody, 1/2000 in 1/20 goat serum and 1 µg/mL DAPI in PBS, 1 hour at RT. After 2 washes in PBS, slides were mounted with aqueous mounting medium. Slides were imaged with a DM5000 microscope (Leica) and Metavue software.

### Statistical analysis

Results were expressed as mean ± SEM and analyzed using GraphPad Prism 5 Software. The Mann–Whitney and one-way ANOVA tests were used to compare data sets. Statistical significance was set at *P* < 0.05.

## Electronic supplementary material

Supplementary figures
