# Supplementary material for: Oxidative damage and impairment of protein quality control systems in keratinocytes exposed to a volatile organic compounds cocktail
Source: Sci Rep. 2017 Sep 6;7:10707. doi: 10.1038/s41598-017-11088-1 (PMC5587662; doi:10.1038/s41598-017-11088-1)

**Oxidative damage and impairment of protein quality control systems in keratinocytes exposed to a volatile organic compounds cocktail.**

Marlène Dezest [1], Mickael Le Bechec [1], Laurent Chavatte [1], Valérie Desauziers [2], Benoît Chaput [3], Jean-Louis Grolleau [3], Pascal Descargues [4], Carine Nizard [5], Sylviane Schnebert [5], Sylvie Lacombe [1], Anne-Laure Bulteau*[1,6] orcid.org/0000-0002-3315-5100

**S.1. Effect of different doses of VOC exposure on keratinocytes viability.**

Effect of combined VOC exposure on keratinocytes viability. Primary keratinocytes prepared from plastic surgery from a 30 year old healthy female donor were exposed to VOCs (toluene, hexane, acetaldehyde, formaldehyde, acetone, at 0.8 or 4 ppmV, each) for 4 hours. Cells were stained with Annexin V-FITC and PI and analyzed by flow cytometry 24 hours after VOC exposure. Percentage of apoptotic cells (Annexin-PI positive) are shown by histogram.

**S.2. Effect of VOC exposure on skin explant viability.**

**A.** Hematoxylin and eosin staining. NativeSkin models from a 33 year old donor female were cultured for 4 days with NativeSkin culture medium, exposed or not to VOCs (toluene, hexane, acetaldehyde, formaldehyde, acetone, 80 ppmV, each) for 4 hours every day. 24 hours after the last VOC exposure, samples were fixed in formalin and embedded in paraffin wax. 5 µm skin cross-sections were stained with hematoxylin eosin. Scale bar is 100 µm. B. Cell proliferation assessment. 5 µm skin cross-sections were immuno-stained for Ki-67. All pictures are representative of the whole sample. Scale bar is 100 µm. C. Cell viability measured with MTT. NativeSkin® models from two different donors (30, and 33 year old) females were cultured for 4 days with NativeSkin® culture medium, exposed or not to VOCs (toluene, hexane, acetaldehyde, formaldehyde, acetone, 80 ppmV, each) for 4 hours every day. 24 hours after the last VOC exposure, MTT assay was carried out. D. Histological scores based on the cumulative counting of the cell anomalies identified from the Hematoxylin and eosin staining (spongiosis, pyknotic nuclei, epidermal detachment). Data, mean ± SEM from 4 skin explants from two different donors (30, and 33 year old) females were used. ** $P < 0.01$.

Supplementary Figure 1

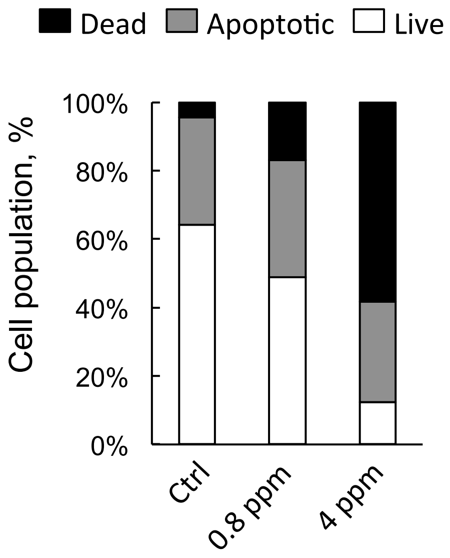

Supplementary Figure 2

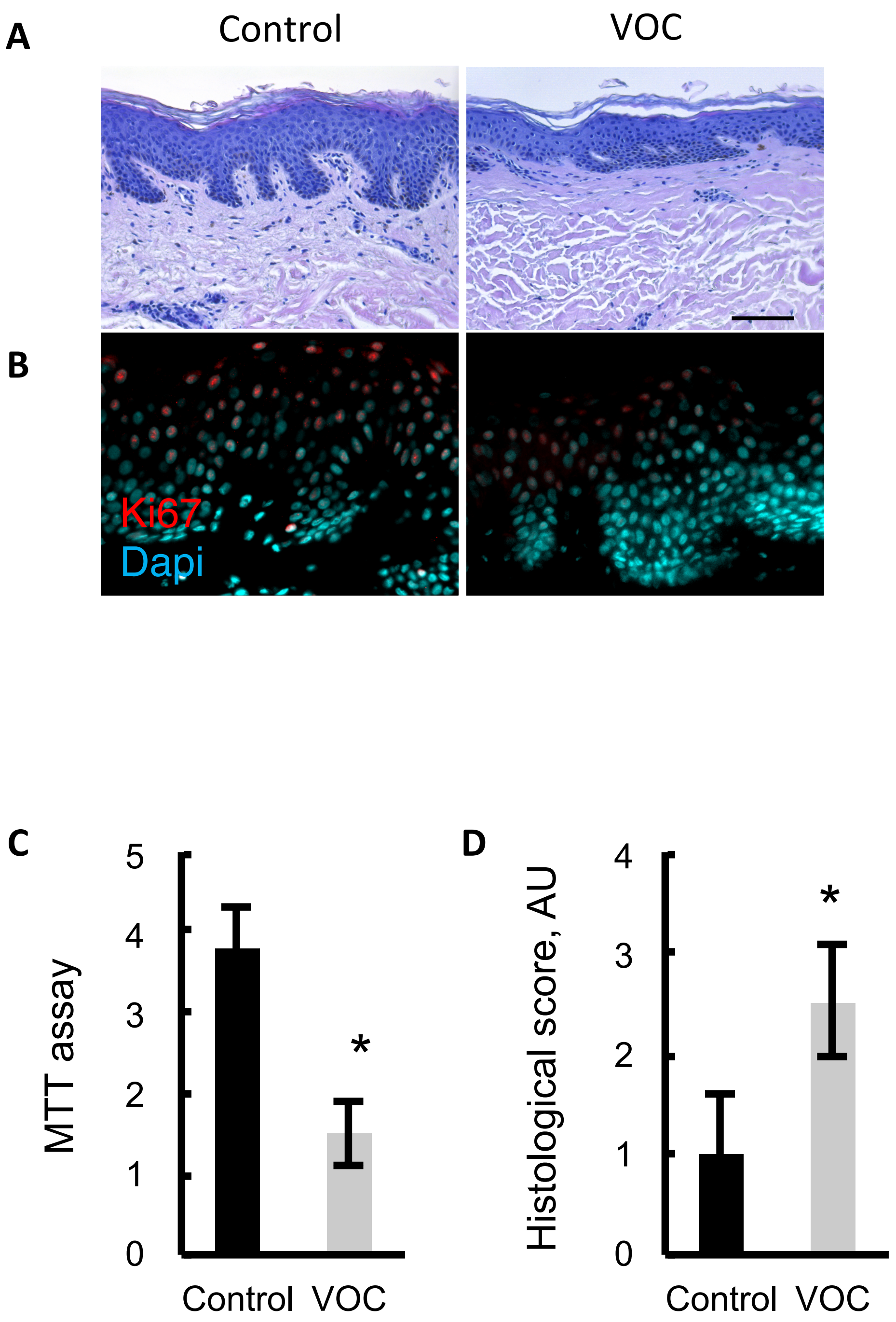

Figure 2D

Respiratory chain complex

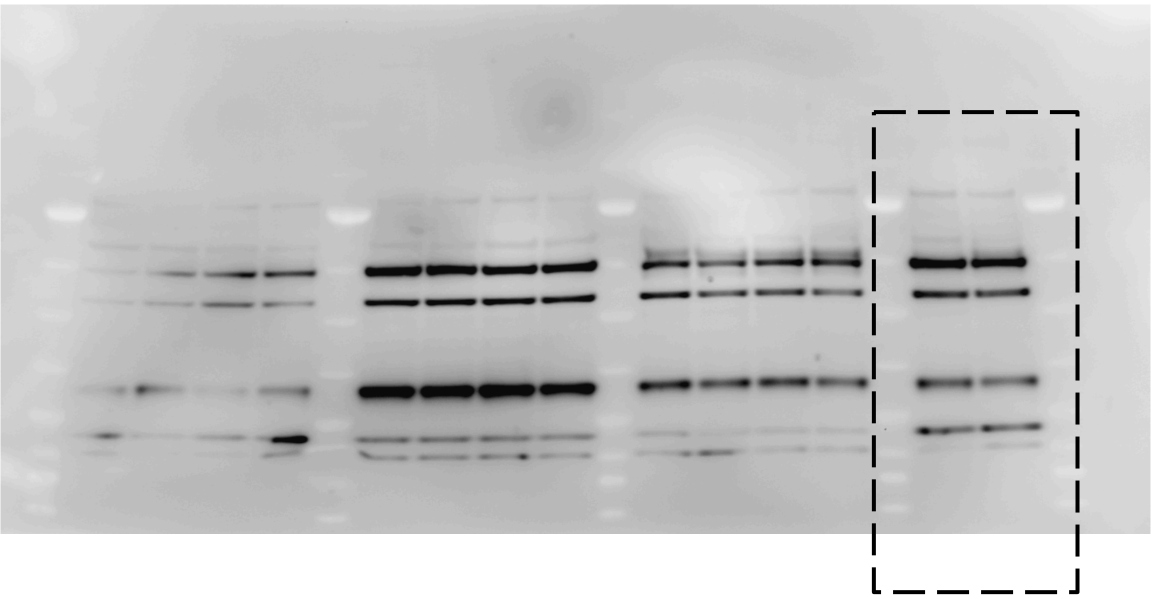

Actin

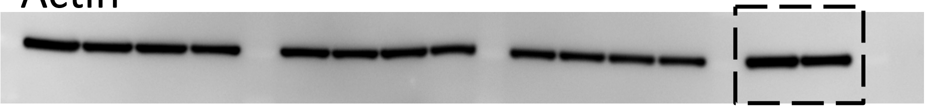

Figure 3

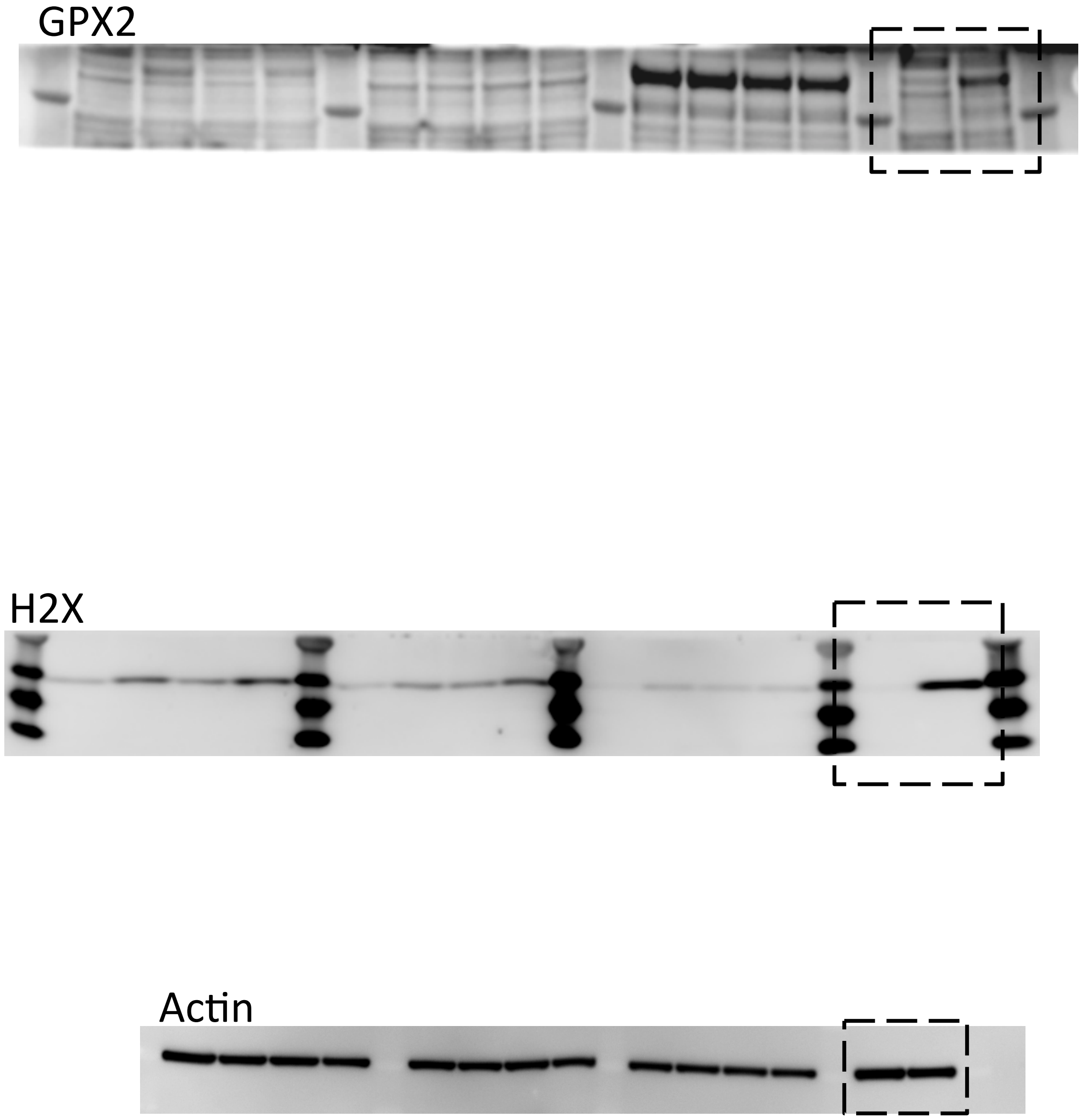

Figure 4

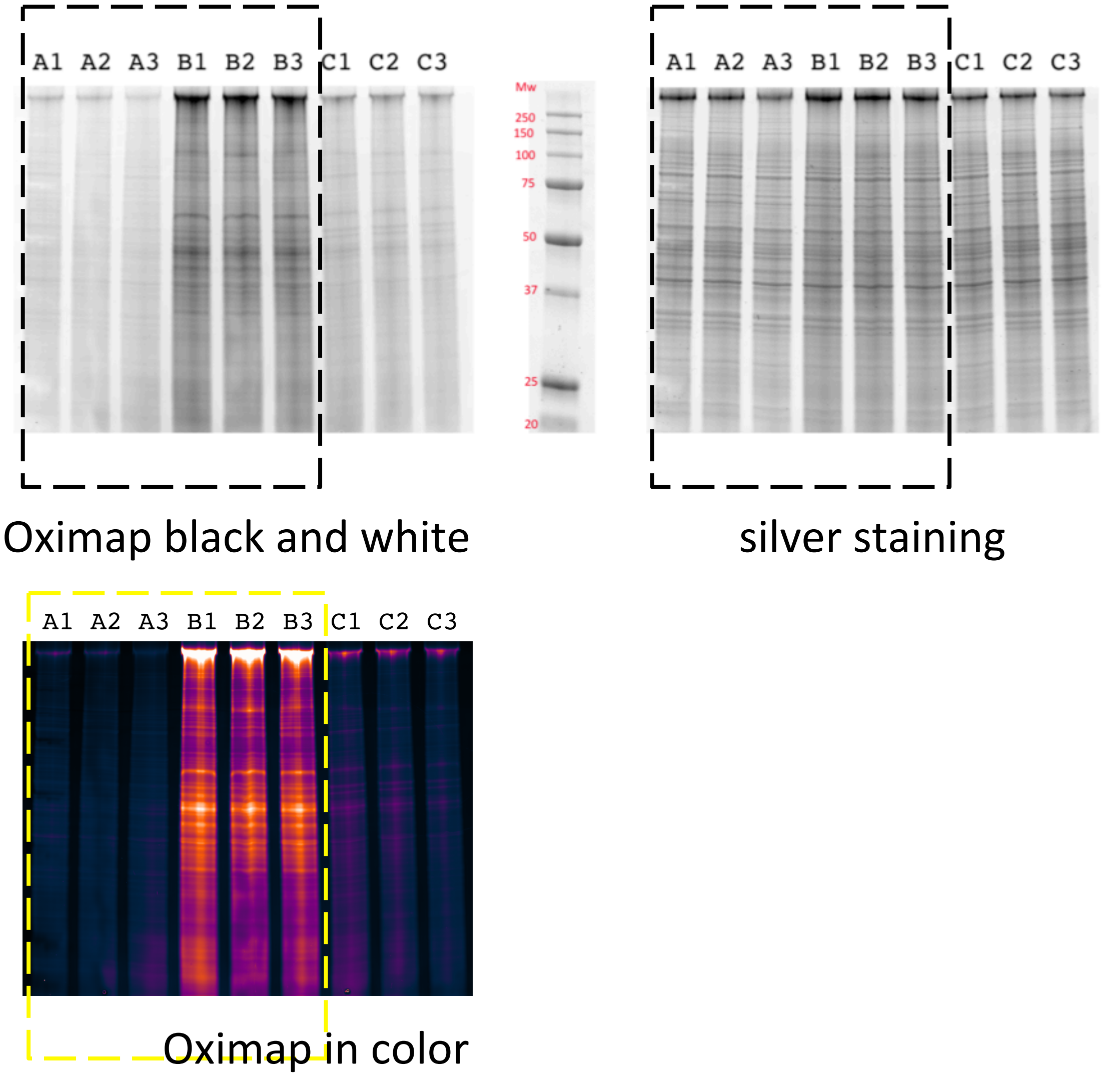
A1 A2 A3 B1 B2 B3 C1 C2 C3
Mw
250
150
100
75
50
37
25
20
A1 A2 A3 B1 B2 B3 C1 C2 C3
Oximap black and white
silver staining
A1 A2 A3 B1 B2 B3 C1 C2 C3
Oximap in color

Figure 5B

Proteasome

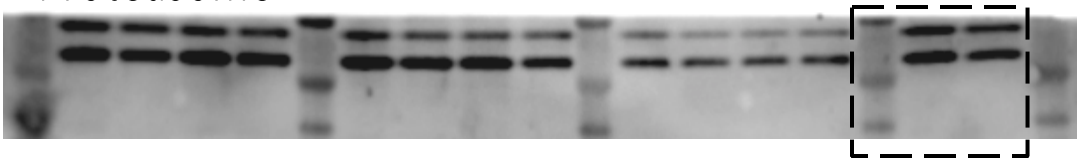

Figure 5E

Lon

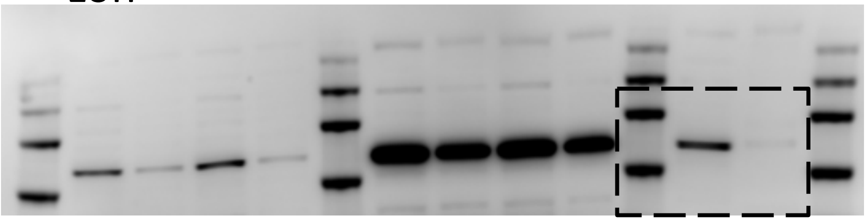

Actin

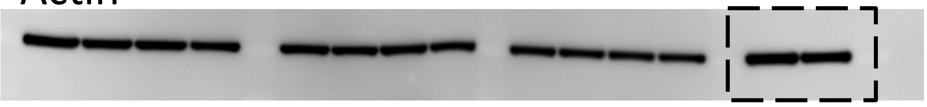

Supplement: Supplementary file 1 — Supplementary figures [file 41598_2017_11088_MOESM1_ESM.pdf]
